# Concomitant use of direct oral anticoagulants and aspirin versus direct oral anticoagulants alone in atrial fibrillation and flutter: a retrospective cohort

**DOI:** 10.1186/s12872-020-01509-x

**Published:** 2020-06-01

**Authors:** Ahmad Said, Scott Keeney, Marsel Matka, Adam Hafeez, Julie George, Alexandra Halalau

**Affiliations:** 1 Internal Medicine Department, Beaumont Health, 44201 Dequindre Rd, Troy, MI 48085 USA; 2 Clinical Instructor, Oakland University William Beaumont School of Medicine, Rochester, MI USA; 3 Internal Medicine Department, Allegheny Health Network Saint Vincent Hospital, Erie, PA USA; 4 Cardiology Division, St Luke’s University Health Center, Bethlehem, PA USA; 5 Cardiology Division, University of Florida, Gainesville, FL USA; 6 Biostatistician, Beaumont Health, Royal Oak, MI USA; 7 General Internal Medicine Division, Beaumont Health, Royal Oak, MI USA; 8 Oakland University William Beaumont School of Medicine, Rochester, MI USA

**Keywords:** Direct oral anticoagulant, Aspirin, Atrial fibrillation, Atrial flutter, Major adverse cardiac events, Bleeding, Harm

## Abstract

**Background:**

The benefit of combining aspirin and direct oral anticoagulants on the reduction of cardiovascular events in atrial fibrillation or flutter is not well studied. We aimed to assess whether concurrent aspirin and direct oral anticoagulant therapy for atrial fibrillation or flutter will result in less coronary, cerebrovascular and systemic ischemic events compared to direct oral anticoagulant therapy alone.

**Methods:**

Retrospective study of adult patients between 18 and 100 years old who have nonvalvular atrial fibrillation or flutter and were started on a direct oral anticoagulant (apixaban, rivaroxaban, or dabigatran), between January 1, 2010 and September 1, 2015 within the Beaumont Health System. Exclusions were history of venous thromboembolic disease and use of other antiplatelet therapies such as P2Y12 inhibitors. Patients were classified into two groups based on concurrent aspirin use and observed for a minimum of 2 years. Primary outcome was major adverse cardiac events, defined as acute coronary syndromes, ischemic strokes, and embolic events. Secondary outcomes were bleeding and death.

**Results:**

Six thousand four patients were in the final analysis, 57% males and 80% Caucasians, median age 71, interquartile range (63–80). The group exposed to aspirin contained 2908 subjects, and the group unexposed to aspirin contained 3096 subjects. After using propensity scores to balance the baseline characteristics in both groups, the analysis revealed higher rate of major adverse cardiac events in the exposed group compared to the unexposed group, (HR 2.11, 95% CI (1.74–2.56)) with a number needed to harm of 11 (95% CI [9–11]). The rate of bleeding was also higher in the exposed group, (HR 1.30, 95% CI (1.11–1.52)). The rate of death was not statistically different between the groups, (HR 0.87, 95% CI (0.61–1.25)).

**Conclusions:**

In this observational analysis of patients with atrial fibrillation and flutter, the concomitant use of direct oral anticoagulants and aspirin was associated with an increased risk of both major adverse cardiac and bleeding events when compared to the use of direct oral anticoagulants alone. These findings underscore the potential harm of this combination therapy when used without a clear indication.

## Background

Atrial fibrillation (AF) is the most common cardiac arrhythmia, with a prevalence of 1% in the United States [1]. Atrial flutter (AFL) is less common, with an estimated incidence of 200,000 cases per year in the United States [2]. AF and AFL are independently associated with increased mortality and morbidity, including stroke, cardiomyopathy, frequent hospitalizations, and cognitive decline. In the Framingham Heart Study cohort, AF was associated with an increase in the risk of mortality to a 1.5-fold in men and 1.9-fold in women [3]. ORBIT-AF demonstrated that 31% of patients with AF had one or more hospitalizations per year [4].

Oral anticoagulation (OAC) based on stroke risk stratification with CHA2DS2-VASc scores has been the mainstay of stroke prevention therapy. OAC can be accomplished with the vitamin K antagonist (VKA) warfarin or one of the direct oral anticoagulants (DOACs) apixaban, edoxaban, dabigatran, or rivaroxaban. Before patients are diagnosed with AF or AFL, a significant number of them already take aspirin (ASA) for either primary or secondary prevention of cardiovascular disease. Apart from acute coronary syndrome (ACS) and percutaneous coronary or vascular interventions, there is no clear, evidence-based threshold to continue or add ASA for primary or secondary prevention of major adverse cardiovascular events (MACE) in the setting of AF or AFL treated with OAC. The 2014 American Heart Association/American College of Cardiology/Heart Rhythm Society practice guidelines and its 2019 focused update for the management of AF do not provide any specific recommendation on concurrent DOAC+ASA use for primary or secondary prevention [5, 6]. The most recent 2016 European Society of Cardiology guidelines emphasize that the use of combination OAC with antiplatelets is contraindicated because of harm (Class III) as this increases bleeding risk and should be avoided in AF patients without another indication for antiplatelet therapy [7–9]. Therefore, there is inconclusive evidence to guide physicians on when to continue or to add ASA therapy in patients with AF/AFL using DOACs.

### Objectives

We hypothesize that the concurrent use of DOACs plus ASA in individuals with AF or AFL will result in lower rates of MACE when compared to DOAC use alone.

## Methods

### Study design

This observational retrospective cohort study aims to compare the effects of combining a DOAC with ASA versus DOAC therapy alone on MACE, bleeding and death in patients with AF or AFL.

### Setting

The study took place at Beaumont Health System, the largest not-for profit health organization in Southeast Michigan. Beaumont Health’s electronic health record (Epic system, Verona, WI, USA) was queried between January 1, 2010 and March 1, 2017 to identify the study population.

### Participants

Patients encountered in any of the inpatient, outpatient or emergency room settings were considered eligible if they were adults between 18 and 100 years of age with documented AF or AFL and taking one of the following DOACs: apixaban, rivaroxaban, or dabigatran. Any classification of AF was accepted (i.e. paroxysmal, persistent, permanent), regardless of the control strategy (i.e. rhythm, rate) and irrespective of previous procedural interventions (i.e. ablation, cardioversion). Patients with valvular AF (i.e. in the setting of rheumatic mitral stenosis or prosthetic valves) were excluded. Other exclusion criteria were a history of venous thromboembolic disease (VTE) such as deep vein thrombosis (DVT) or pulmonary embolism (PE), in order to exclude patients with competing reasons for anticoagulation. Patients who were taking different antiplatelets such as P2Y12 inhibitors (i.e. clopidogrel, prasugrel or ticagrelor) were also excluded. The International Classification of Diseases, Ninth and Tenth Revisions, Clinical Modification (ICD-9-CM & ICD-10-CM) codes were used to identify the study patients. The cohort was divided into two groups: individuals taking ASA in addition to a DOAC (exposed group) and patients taking a DOAC without ASA (unexposed group). The indication for ASA use and the dosage was not assessed. All subjects were observed for a minimum of 2 years. The outcomes of interest were identified by querying hospital readmission diagnoses, inpatient diagnoses, discharge diagnoses, and active problem list using ICD-9-CM and ICD-10-CM codes.

### Variables

The primary outcome is the composite major adverse cardiac events (MACE) defined as (1) ischemic cerebrovascular events including stroke and transient ischemic attack, (2) systemic embolism to any vascular territory outside the central nervous system, (3) and acute coronary syndromes (ACS) including unstable angina, non-ST elevation and ST elevation myocardial infarctions. Secondary outcomes are all cause mortality and bleeding, defined as any bleeding event leading to hospital presentation or admission; the severity of bleeding was not addressed as all events were considered severe if they prompted hospital presentation. Only the first event was analyzed and patients who experienced subsequent events were censored after experiencing any of the above outcomes.

The variables assessed included: patient age, gender, and race, in addition to multiple comorbidities that can have an effect on the risk of developing cardiovascular disease and bleeding. We also calculated a CHADS-VASc score for each patient (a validated clinical prediction tool for estimating the risk of stroke in non-rheumatic atrial fibrillation) and the HASBLED score for each patient (a validated scoring system developed to assess 1-year risk of major bleeding in patients taking anticoagulation with atrial fibrillation).

### Data sources/measurement

The data including study population, variables and outcomes was extracted from the electronic health record through Toad Data Point with query of medical & surgical histories, active medication lists, problem lists, procedure notes, hospital discharge diagnoses, and hospital primary diagnoses.

### Bias

All patients identified in Beaumont’s healthcare database who met the inclusion criteria were included in the study in an attempt to minimize selection bias. Propensity scores were calculated for baseline characteristics and used to inversely weigh all observations in an attempt to achieve balance in the treatment groups and minimize confounders.

In an effort to minimize information (measurement) bias, automated reports of patient data and outcomes were generated by an individual who was not involved in the study protocol or statistical analysis. Covariates, outcomes, and baseline characteristics were obtained in a standardized fashion without knowledge of the patient groups. Moreover, regular meetings with the data collectors were held to ensure variables were obtained in a consistent fashion, thus minimizing inter-observer variability. Additionally, our biostatistician was not involved in the study design and data collection or interpretation. Researcher bias was limited via strict adherence to the study protocol. Finally, the impact of residual confounding was minimized by adjusted analysis for known confounders; however, the potential for unidentified or unknown confounders exists.

### Statistical methods

Differences in baseline characteristics between the two treatment groups (DOAC+ASA and DOAC only) were compared using the χ^2^ test for categorical variables and the Student unpaired *t* test for continuous variables, as appropriate.

Before analyzing outcomes, a propensity score was calculated for each patient in the analysis dataset. Propensity score was defined as the estimated probability of being “treated” (which for this study means having index treatment of “DOAC+ASA”) as a function of covariates. The following covariates were included in the calculation: sex, race, age, tobacco use, body mass index (BMI), CHADS-VASc score, history of anemia, coronary artery disease (CAD), cancer, congestive heart failure (CHF), chronic kidney disease (CKD), chronic obstructive pulmonary disease (COPD), diabetes mellitus (DM), gastrointestinal (GI) bleed, myocardial infarction (MI), obstructive sleep apnea (OSA), peptic ulcer disease (PUD), stroke, peripheral vascular disease, baseline use of non-steroidal anti-inflammatory drugs (NSAID), protein pump inhibitors (PPI), statins, angiotensin converting enzyme inhibitors (ACEi), and beta blockers.

Propensity score was then used to balance the treatment groups in terms of covariate distributions by weighting each observation by the inverse probability of treatment. Additionally, because there were a few observations with extremely large weights, we standardized the weights by the actual (sample) proportion of treated. Weighting results in a synthetic sample in which the distribution of baseline covariates is independent of treatment. Once balance in covariates was achieved, weighted data was used for subsequent analyses. A Cox proportional hazards model was employed to estimate hazard ratios for each of the three outcomes (MACE, bleeding, and death). Treatment was included in all models, and adjusted for sex, race, age, tobacco use, body mass index (BMI), CHADS-VASc score, history of anemia, coronary artery disease (CAD), cancer, congestive heart failure (CHF), chronic kidney disease (CKD), chronic obstructive pulmonary disease (COPD), diabetes mellitus (DM), gastrointestinal (GI) bleed, myocardial infarction (MI), obstructive sleep apnea (OSA), peptic ulcer disease (PUD), stroke, peripheral vascular disease, baseline use of non-steroidal anti-inflammatory drugs (NSAID), protein pump inhibitors (PPI), statins, angiotensin converting enzyme inhibitors (ACEi), and beta blockers. HASBLED scores were not included in the calculation of propensity scores or in the adjusted models because aspirin use automatically adds a point to the score, thus none of the subjects in the exposed group would have had a score of zero.

Time-to-event curves comparing treatment groups for MACE, bleeding and death were created using predicted probabilities of event-free survival from the adjusted Cox regression models. Number Needed to Harm (NNH) was calculated using the predicted MACE rates from the weighted and adjusted Cox proportional hazards models.

All analyses were conducted using SAS version 9.4**,** (SAS Institute, Cary, NC). Statistical significance was assumed at a *p-*value < 0.05. All tests were 2-sided.

## Results

### Participants

**Fig. 1:**
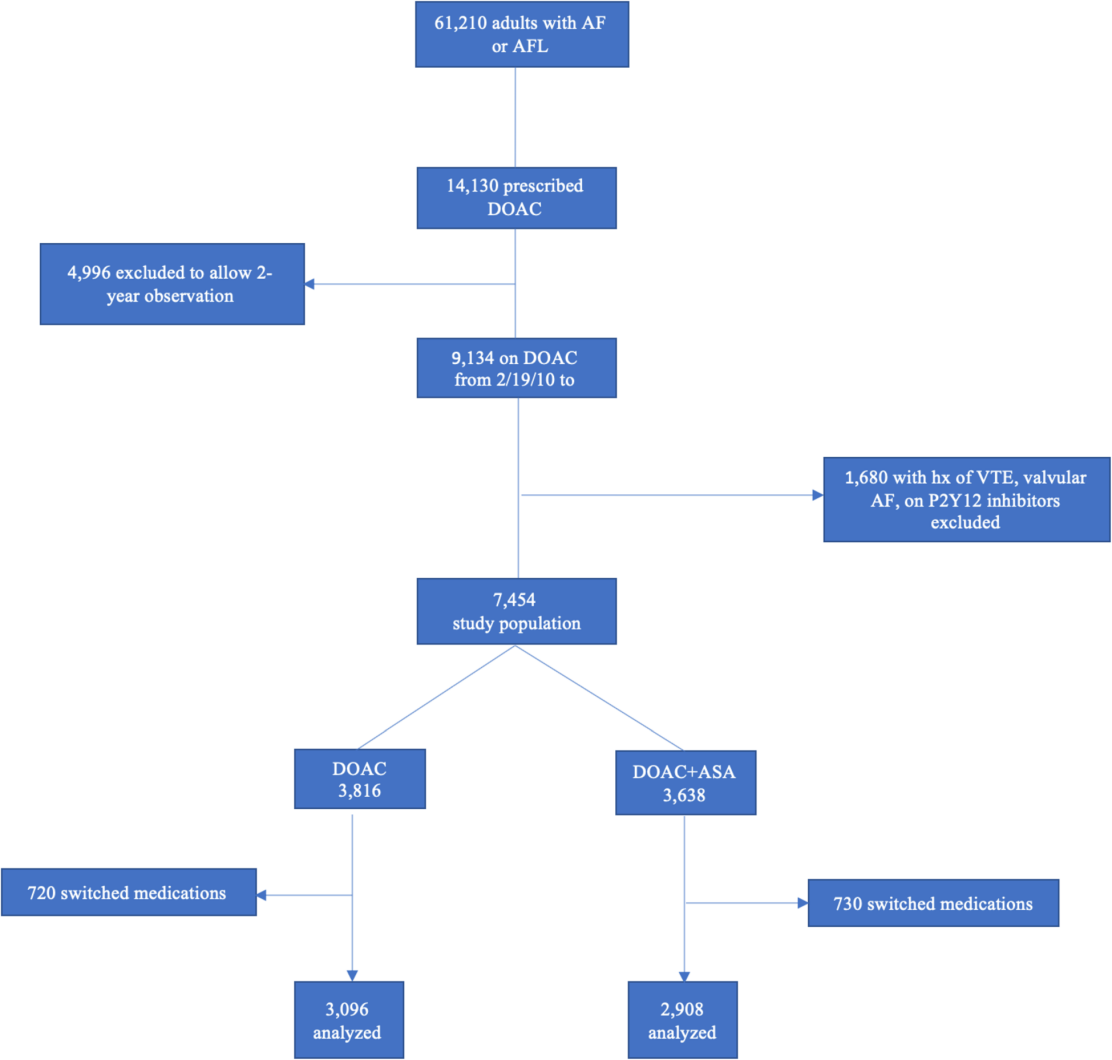
Study Flowchart

Sixty-one thousand two hundred ten patients with a diagnosis of AF or AFL were identified between January 1, 2010 and March 1, 2017, of which 14,130 were prescribed a DOAC. As the minimum observation period was set at 2 years, 4996 patients were excluded because they were prescribed a DOAC after September 1, 2015. After the exclusion criteria were applied to the remaining 9134 patients, 7454 patients were left and subsequently divided into two groups as follows: 3638 individuals on a DOAC+ASA (exposed) and 3816 individuals on a DOAC alone (unexposed). During event analysis, it was discovered that 730 patients in the exposed group and 720 patients in the unexposed group were no longer in the group they started the study in due to medication change and those patients were not included in the final analysis. There were 13 patients with missing BMI information but those were still included in the final analysis. Therefore, 2908 subjects in the exposed group and 3096 in the unexposed group were analyzed (Fig. 1).

### Descriptive data

The mean age for the study population was 70.5, standard deviation (SD) of 12.3. The median age was 71. Out of the total cohort, 57% were males and 80% Caucasians. Mean BMI was 30.5 (SD = 7.3) and the median BMI was 29.2. Tobacco use was documented in 45% of patients. The mean CHADS-VASc score was 2.9 (SD = 1.8) and the median was 3%. Out of all patients, 9% had a history of stroke, 13% had CAD, 3% had a history of GI bleed and 5% had CKD. 11% of patients were taking NSAIDs and 29% were taking PPI. The DOAC+ASA group contained 2908 subjects (48%), and the DOAC only group contained 3096 (52%). The median time of follow up was 41.2 months. After propensity score weighting, no differences were found in the baseline characteristics of the two groups (Table 1).

**Table 1:** Baseline (demographic and clinical) characteristics of the total study population and of the two groups DOAC only and DOAC+ASA

	N	6004	DOAC Only	DOAC + Aspirin	*p-value*	DOAC Only	DOAC + Aspirin	*p-value*
			3096	2908		3096	2908	
		Overall	By Treatment (unwtd)			By Treatment (wtd)		
**Age***(mean, std dev)*		70.5, 12.3	69.8, 12.5	71.3, 12.1	*< 0.001*	70.4, 12.1	70.3, 12.6	*0.746*
*Median*		71	71	72		71	71	
*IQ range*		63–80	62–79	64–80		63–79	62–80	
**Female**	2597	43%	42%	45%	*0.047*	43%	43%	*0.759*
**Race**					*0.037*			*0.998*
Black	390	6%	6%	7%		6%	7%	
White	4797	80%	80%	80%		80%	80%	
Other	411	7%	7%	6%		7%	7%	
Unknown	406	7%	7%	6%		7%	7%	
**Tobacco**	2716	45%	35%	56%	*< 0.001*	47%	48%	*0.653*
**BMI***(mean, std dev)*		30.5, 7.3	30.4, 7.2	30.6, 7.5	*0.304*	30.7, 7.4	30.4, 7.3	*0.135*
*Missing*		13	9	4				
*Median*		29.2	29.0	29.3		29.3	29.0	
*IQ range*		25.7–34.1	25.6–33-9	25.7–34.3		25.8–34.1	25.5–33.9	
**CHADS VASc Score** *(mean, std dev)*		2.9, 1.8	2.5, 1.7	3.3, 1.8	*< 0.001*	2.9, 1.7	2.9, 1.8	*0.763*
*Median*		3	2	3		3	3	
*IQ range*		2–4	1–4	2–4		2–4	2–4	
**HASBLED Score** *(mean, std dev)*		1.22, 0.84	0.67, 0.66	1.81, 0.57		NA	NA	
**CHF**	828	14%	10%	17%	*< 0.001*	14%	14%	*0.769*
**HTN**	3758	63%	53%	73%	*< 0.001*	63%	64%	*0.459*
**DM**	1145	19%	14%	25%	*< 0.001*	19%	19%	*0.734*
**Stroke**	518	9%	7%	11%	*< 0.001*	9%	9%	*0.923*
**Vascular Disease**	1349	22%	16%	30%	*< 0.001*	22%	22%	*0.794*
**Anemia**	481	8%	6%	10%	*< 0.001*	8%	8%	*0.877*
**CAD**	804	13%	10%	17%	*< 0.001*	13%	13%	*0.901*
**Cancer**	925	15%	14%	17%	*0.003*	16%	16%	*0.609*
**CKD**	288	5%	4%	6%	*< 0.001*	5%	5%	*0.968*
**COPD**	528	9%	6%	11%	*< 0.001*	9%	9%	*0.882*
**GI Bleed**	176	3%	3%	3%	*0.908*	3%	3%	*0.971*
**MI**	347	6%	3%	9%	*< 0.001*	6%	6%	*0.685*
**OSA**	325	5%	5%	6%	*0.045*	5%	5%	*0.905*
**PUD**	167	3%	3%	3%	*0.422*	3%	3%	*0.970*
**Index NSAID**	683	11%	9%	13%	*< 0.001*	13%	13%	*0.925*
**Index PPI**	1733	29%	21%	37%	*< 0.001*	30%	31%	*0.583*
**Index Statin**	2769	46%	32%	61%	*< 0.001*	47%	48%	*0.613*
**Index ACE**	1489	25%	19%	31%	*< 0.001*	26%	26%	*0.686*
**Index Beta B**	1323	22%	15%	29%	*< 0.001*	23%	23%	*0.951*

Abbreviations: *unwtd* unweighted, *wtd* weighted, *BMI* body mass index, *CHF* congestive heart failure. *NA* not applicable, *HTN* hypertension, *DM* diabetes mellitus, *CAD* coronary artery disease, *CKD* chronic kidney disease, *COPD* chronic obstructive pulmonary disease, *GI* gastrointestinal, *MI* myocardial infarction, *OSA* obstructive sleep apnea, *PUD* peptic ulcer disease, *NSAID* nonsteroidal anti-inflammatory drug, *PPI* proton pump inhibitors, *ACEi* angiotensin converting enzyme inhibitors, *Beta B* beta blockers

### Outcome data

Of the 6004 patients in the final analysis, 110 had ACS (1.8%), 367 had ischemic CVA’s (6.1%), and 10 had other embolic events (0.2%). Six hundred twelve patients presented to the hospital with a bleeding event (10.2%). Death occurred in 122 patients (2.0%). Four thousand seven hundred eighty-three patients did not have any events during the observation period (79.7%) (Table 2).

**Table 2:** Number and rate of events before and after propensity weighting

	Overall (*N* = 6004)			DOAC + Aspirin (*N* = 2908)			DOAC only (*N* = 3096)		
	Number of occurrence	Actual rate	Weighted rate	Number of occurrence	Actual rate	Weighted rate	Number of occurrence	Actual rate	Weighted rate
ACS	110	1.8%	1.6%	89	3.1%	2.6%	21	0.7%	0.6%
Bleeding	612	10.2%	10.3%	365	12.6%	11.4%	247	8.0%	9.3%
Death	122	2.0%	2.1%	59	2.0%	1.9%	63	2.0%	2.2%
Emboli	10	0.2%	0.1%	9	0.3%	0.3%	1	0.0%	0.0%
Stroke	367	6.1%	5.9%	250	8.6%	7.4%	117	3.8%	4.6%
None	4783	79.7%	80.0%	2136	73.5%	76.4%	2647	85.5%	83.3%

### Main results

**Fig. 2:**
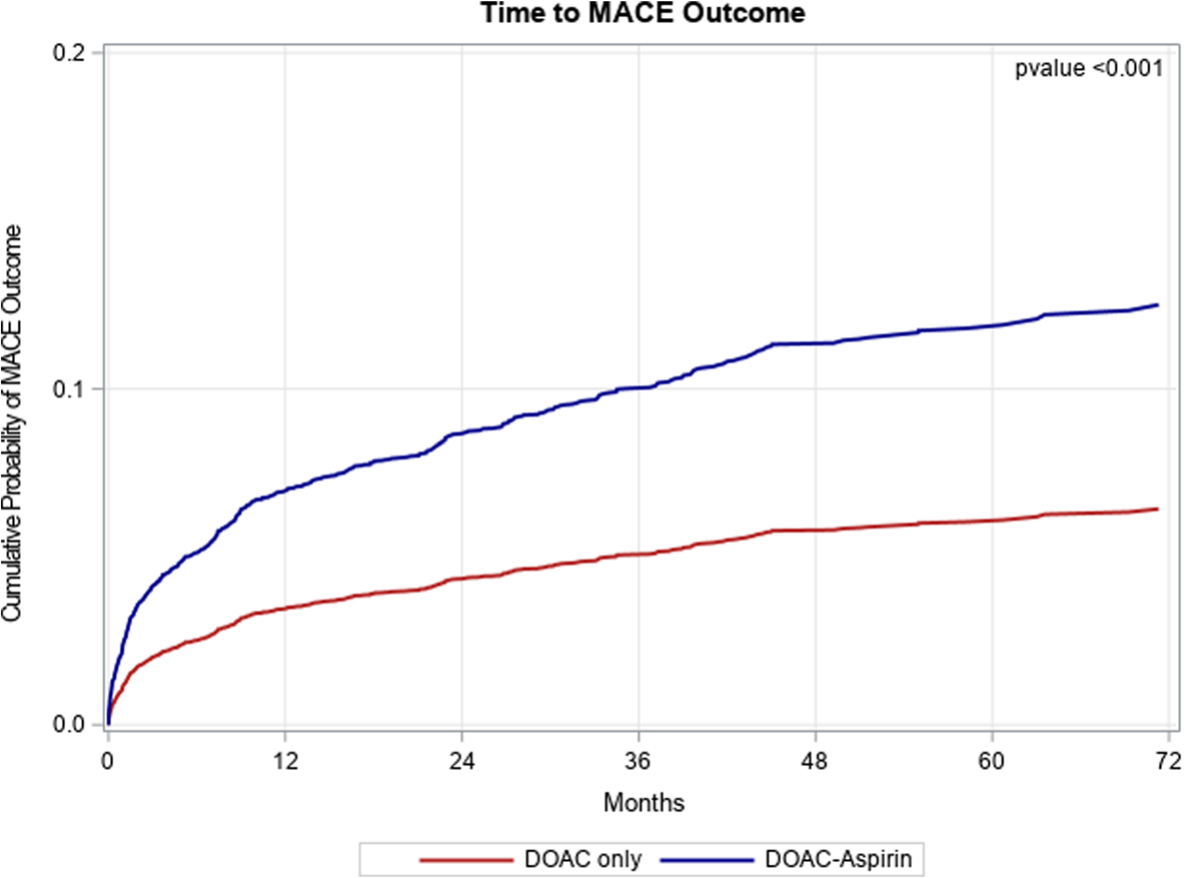
Cumulative incidence of MACE in the study population. The red line represents the DOAC only group and the blue line represents the DOAC+ASA group. Abbreviations: MACE, major adverse cardiac events; DOAC, direct oral anticoagulants; ASA, aspirin

Following propensity weighting and adjusting for all baseline characteristics detailed in Table 1, the rates of ACS and ischemic CVA in the exposed vs unexposed groups were 2.6 and 7.4% vs 0.6 and 4.6%, respectively (Table 2). MACE occurred more in the exposed group (14.6%) compared to the unexposed group (5.4%), adjusted hazard ratio (HR) 2.11, 95% confidence interval (1.74, 2.56) (Fig. 2). The number needed to harm is 11, 95% confidence interval (9, 13).

**Fig. 3:**
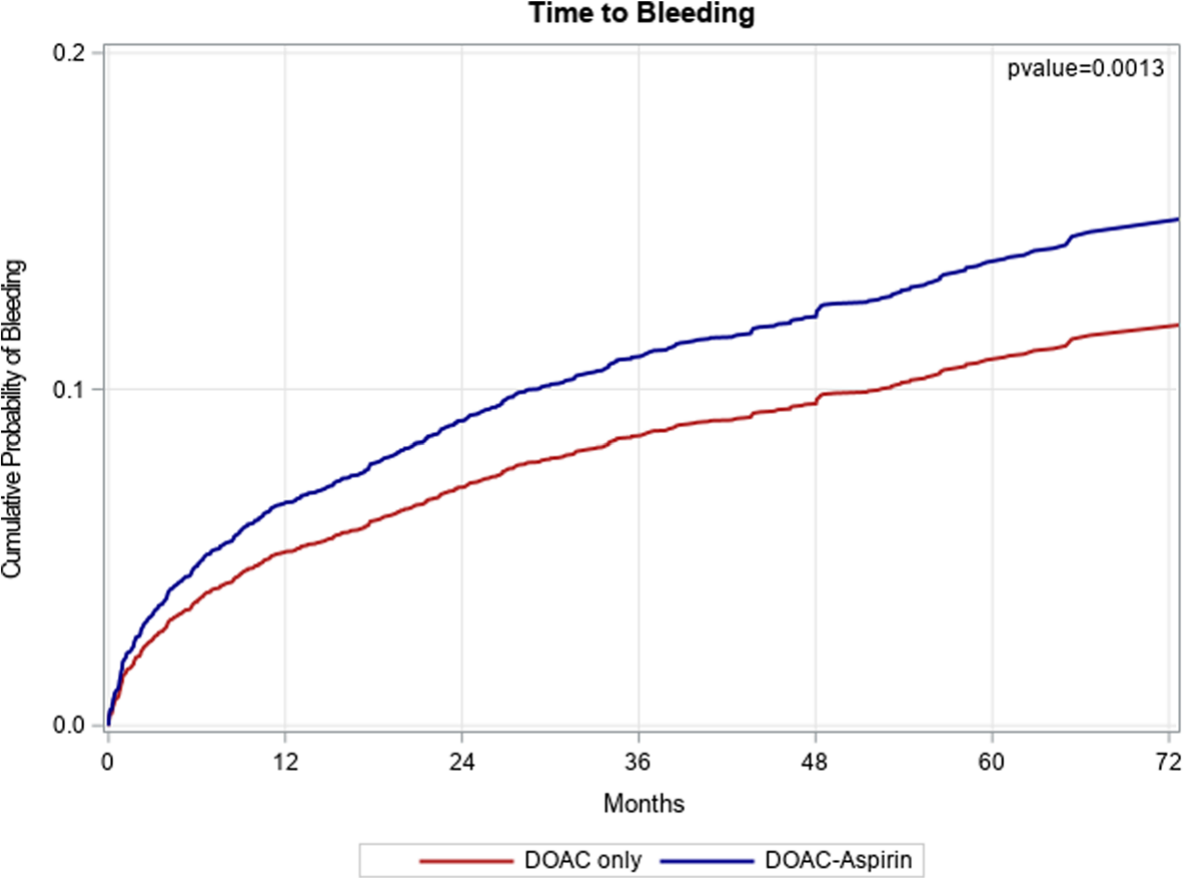
Cumulative incidence of bleeding in the study population. The red line represents the DOAC only group and the blue line represents the DOAC+ASA group. Abbreviations: DOAC, direct oral anticoagulants; ASA, aspirin

**Fig. 4:**
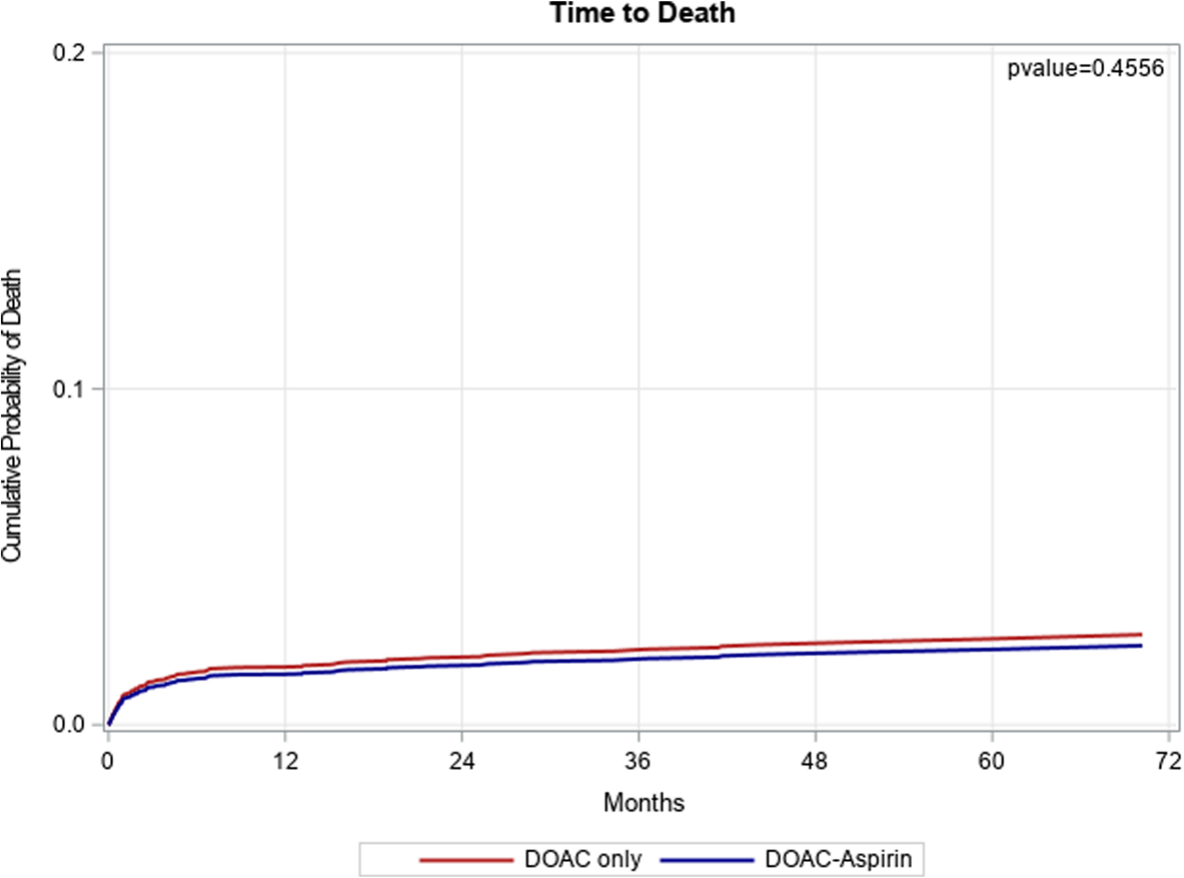
Cumulative incidence of death in the study population. The red line represents the DOAC only group and the blue line represents the DOAC+ASA group. Abbreviations: DOAC, direct oral anticoagulants; ASA, aspirin

With respect to secondary outcomes, bleeding occurred more in the exposed group (19.3%) compared to the unexposed group (11.8%), adjusted HR 1.30, 95% CI (1.11, 1.52) (Fig. 3). Death rates were not statistically different between the two groups (2.6% vs 2.5%), adjusted HR 0.87, 95% CI (0.61, 1.25) (Fig. 4).

## Discussion

Our study evaluated patients with AF or AFL on a DOAC and aimed to assess the benefits and harms of concomitant ASA therapy. The analysis demonstrated higher rates of composite MACE in patients taking the combination of ASA + DOAC when compared to similar patients taking a DOAC alone. Additionally, individual rates of ACS, ischemic CVA, and non-central nervous system embolic events were each greater in the group exposed to ASA. These results correlate with Kumar et al’s meta-analysis that pooled data from the four DOAC randomized controlled trials in which 33.4% of patients were already taking ASA or another antiplatelet drug. Their comparison detected a signal towards higher thromboembolic rates among DOAC users also on ASA/antiplatelet drugs when compared with DOAC alone [10]. In Lemesle et al’s analysis of data from the REACH registry, patients with AF and stable CAD who were on a VKA + antiplatelet were compared to those only on a VKA, and the study yielded no statistically significant difference in MACE or death between the two groups [11].

Although our study adjusted for a wide range of baseline comorbidities that can affect the outcomes of interest, it is quite challenging to qualify the severity of these individual variables and compare them between the two groups. Hence, one possible explanation for the higher occurrence of cardiovascular events among patients in the exposed group (DOAC+ASA) is the possibility that certain comorbid conditions, such as CAD, were more extensive in those patients as compared to patients taking a DOAC alone.

Our analysis also identified the combination therapy group experienced more bleeding events that prompted hospital presentations. These findings are similar to previously published literature in that both Kumar and Lemesle detected higher rates of bleeding in subjects taking the combination of a DOAC+antiplatelet compared to a DOAC alone and in subjects taking a VKA + antiplatelet compared to a VKA alone, respectively. Additionally, Steinberg et al. studied the ORBIT-AF registry population and found a significantly higher rate of major bleeding and bleeding hospitalizations in patients on combined OAC + ASA therapy compared to those on OAC alone [4].

This study is limited by unknown confounding variables inherently present in a retrospective, observational analysis including non-randomly assigned treatment groups. Although we attempted to minimize confounding, the data was limited to electronic health record (EHR) documentation and adjudication of variables such as baseline characteristics and outcomes via individual chart review was not performed. As such, the severity of comorbid conditions present at baselines was not investigated. For example, hemoglobin A1c in diabetics, lipid profiles in hyperlipidemic patients, or the burden of atherosclerotic plaques in patients with CAD were not adjudicated or compared between the treatment groups. Only the presence or absence of the disease was included. Additionally, the severity of each bleeding event was not investigated. As mentioned above, the indication for ASA was not ascertained and the specific dose was not analyzed. Finally, it was unclear if patients who did not experience an outcome of interest remained in the same treatment group that they began the study in.

Strengths of this study include a large sample size, thorough data collection to account for pertinent comorbid conditions, medications, and patient characteristics, as well as adjustment and propensity weighting to help ensure the study groups were as similar as possible. Additionally, we allowed a minimum of 2 years of follow up to identify the outcomes. With respect to external validity, we believe that the results of this cohort study are highly generalizable to the target population since we included all adult patients across both outpatient and inpatient encounters with nonvalvular AF/AFL anticoagulated with a DOAC; therefore, the patient population contains a wide range of patient characteristics and comorbid conditions.

Our focus on the use of DOAC and ASA alone (to the exclusion of other anticoagulants and antiplatelets) is what distinguishes our study from many of those previously described. Nonetheless, our data reinforces a similar concept that the combination of anticoagulants and ASA may be responsible for more harm than benefit when the anticoagulant is being utilized for thromboembolic prophylaxis in AF or AFL. This is important given the increasing popularity of DOAC use in patients with AF and AFL, in addition to the widespread use of ASA that is encountered in daily clinical practice. Due to the absence of strong guidelines regarding concomitant use of these agents, the decision is often left to the individual treating clinician.

Recent investigations into the role of ASA for primary prevention of atherosclerotic cardiovascular disease have led to several large meta-analyses and subsequent changes to national guidelines [12] (Arnett et al). With the exception of a decrease in non-fatal ischemic events, in aggregate, these trials did not demonstrate a reduction of MACE while leading to significantly more major bleeding events [13, 14]. This places a greater emphasis on the need to carefully weigh the risks and benefits of initiating and/or continuing ASA therapy, especially in the setting of concurrent DOAC use for AF or AFL.

## Conclusion

In conclusion, patients with AF and AFL prescribed a DOAC are often also treated with ASA. Our results demonstrate that the concomitant use of DOACs and ASA was associated with an increased risk of MACE compared to the use of DOACs alone, as well as an increased risk of bleeding. These findings and previously published data suggest that caution must be taken to identify subjects who would benefit from concurrent ASA + DOAC therapy. We believe that randomized controlled trials are warranted to definitely assess the benefit or harm of such strategies.

## Data Availability

All data generated and analyzed for our study is available upon request and is stored in a secured, encrypted database approved by our institution.

## Abbreviations

AF: Atrial fibrillation
AFL: Atrial flutter
ASA: Aspirin
ACS: Acute coronary syndrome
CAD: Coronary artery disease
CVA: Cerebrovascular accident
CI: Confidence interval
DOAC: Direct oral anticoagulants
EHR: Electronic health record
HR: Hazard ratio
MACE: Major adverse cardiac events
OAC: Oral anticoagulant
VKA: Vitamin K antagonist
BMI: Body mass index
CHF: Congestive heart failure
HTN: Hypertension
DM: Diabetes mellitus
CKD: Chronic kidney disease
COPD: Chronic obstructive pulmonary disease
GI: Gastrointestinal
MI: Myocardial infarction
OSA: Obstructive sleep apnea
PUD: Peptic ulcer disease
NSAID: Nonsteroidal anti-inflammatory drug
PPI: Proton pump inhibitors
ACEi: Angiotensin converting enzyme inhibitors
Beta B: Beta blockers
